# Spindle Cell Lipoma and Pleomorphic Lipoma in the Head and Neck: A Comprehensive Study of Six Cases With Review of Literature

**DOI:** 10.7759/cureus.61029

**Published:** 2024-05-24

**Authors:** Subhash Yadav, Katha Rabade, Swapnil Rane, Asawari Patil, Neha Mittal, Sumankumar Ankathi, Sumeet Gujral, Bharat Rekhi, Munita Bal

**Affiliations:** 1 Department of Surgical Pathology, Tata Memorial Hospital & Advanced Centre for Treatment, Research and Education in Cancer (ACTREC) A Constituent Institution (CI) of Homi Bhabha National Institute, Mumbai, IND; 2 Department of Radiodiagnosis, Tata Memorial Hospital & Advanced Centre for Treatment, Research and Education in Cancer (ACTREC) A Constituent Institution (CI) of Homi Bhabha National Institute, Mumbai, IND

**Keywords:** molecular profile, cd34, larynx, lipoma, spindle cell, head and neck

## Abstract

Background: Spindle cell lipomas (SL) and pleomorphic lipomas (PL) are rare variants of lipomas, occurring predominantly in the head and neck region. Laryngeal SL/PL is very uncommon and causes obstructive symptoms needing immediate intervention. These tumors are often challenging in radiology due to the admixture of elements and the presence of adipose tissue may help in diagnosis. From a surgeon's perspective, understanding the nuances of SL/PL is paramount. Histology is the gold standard for diagnosis; however, it often causes diagnostic challenges in biopsy.

Method: A retrospective review of the clinical and pathologic features of archival cases of SL/PL was performed.

Results: A total of six cases of head and neck region SL/PL were identified. The age of patients ranged from 21 to 58 years and the male-to-female ratio was 5:1. The tumors were distributed in the nape of the neck (n=3), laryngeal region (n=2), and orbit (n=1). Histology in all the cases showed a low-grade neoplasm composed of a variable amount of spindle cells and adipose tissue. The stroma was myxoid in most cases. CD34 was diffusely positive in all the cases.

Conclusion: SLs are a rare and uncommon variant of lipoma with a predilection in the head and neck region. They are low-grade neoplasms with a propensity to recur after years. Having knowledge of this tumor can improve surgical outcomes and better patient care.

## Introduction

Lipomas are benign adipocytic neoplasms composed of mature adipocytes with or without other mesenchymal elements. Many subtypes such as fibrolipoma, myoid lipoma, angiolipoma, spindle cell lipoma (SL), and pleomorphic lipoma (PL) have been described to date [1]. SLs were first described by Enzinger in 1975 [2]. It is now established that SL/PL represents a morphologic spectrum of the same neoplasm. The former is characterized by mature fat, spindle cells, and ropy collagen while PL contains pleomorphic and multinucleated floret cells. These typically arise in the subcutaneous tissue of the posterior neck, upper back, and shoulders [2]. Uncommonly, SL/PL are identified in the subcutis of the extremities, trunk, and head and neck region while isolated cases have been reported in the kidney, vulva, and perianal region [3-5]. Within the head and neck area, SL/PL tends to involve the scalp, face, or oral cavity. Occurrence in the larynx is exceptionally rare with only six cases reported in the literature to date [6-8].

These tumors have a wide morphologic spectrum including the presence of mesenchymal elements and/or pleomorphic cells, which renders the histologic diagnosis of SL/PL extremely challenging and fraught with diagnostic pitfalls. Atypical radiologic picture due to mesenchymal components further augments the diagnostic difficulty. As a result, a diagnosis of lipoma is usually not considered when the tissue is submitted for histopathologic diagnosis. Hence, the onus of diagnosis completely shifts to the pathologist in the majority of cases. Therefore, awareness of the pathologic spectrum and differential diagnostic entities of SL/PL is essential for pathologists to avoid making erroneous diagnoses and subsequent management. Despite their benign nature, the diagnostic nuances associated with SL/PL underscore the importance of accurate identification and appropriate management strategies by surgeons. Herein, we present a series of six cases of SL/PL occurring in the head and neck region (including at rare sites such as vallecula and larynx) and expand on its morphologic spectrum as well as the challenges entailed in its diagnosis and supplement it with a review of literature on this rare entity.

## Materials and methods

This was a retrospective observational study involving a review of existing data over a specific period followed by an analysis of the same without manipulating the variables or the study environment. The study was conducted at the Department of Surgical Pathology, Tata Memorial Hospital, Parel, Mumbai, India. All cases of SL/PL diagnosed from January 2010 to December 2020 at our tertiary care oncology center were retrieved from the archives of the Department of Pathology. The search words included “lipoma”, “spindle lipoma” and “pleomorphic lipoma”. A total of six cases were retrieved from the database reported as “spindle cell lipoma” or “pleomorphic lipoma”.

Hematoxylin and eosin (H&E)-stained slides and immunohistochemistry (IHC) slides were reviewed by two experienced pathologists, and the diagnosis was confirmed as per the WHO classification of soft tissue and bone tumors [1]. Cases with discrepancies between pathologists or cases without IHC results were excluded from the study. The clinical data, including patient demographics, presenting symptoms, physical examination findings, and relevant medical history, were extracted from the hospital's electronic medical records for each case. Additionally, radiological information (MRI and CT scans) was collected to complement the clinical data.

Ethical considerations

No patients were directly or indirectly contacted as part of this study, and only histopathological and clinical details were analyzed without any personal identifiers. The study did not involve any risk to any participants. The data was confidential and anonymized and will not have any impact on the outcomes of the participants. Consent was not sought in view of small sample size, retrospective design, and no/minimum risk to the patients.

Statistical analysis

Descriptive statistics were used to summarize the demographic, clinical, and pathological characteristics of the study cohort. Continuous variables were presented as means with standard deviations or as medians with interquartile ranges, depending on the data distribution. Categorical variables were expressed as frequencies and percentages. Follow-up information was incorporated wherever available.

## Results

A total of six cases of SL/PL were identified in the head and neck region. The clinical details of the patients are summarized in Table 1. The mean age of patients was 34.8 years with a striking male-to-female ratio of 5:1. There were three cases of SL/PL in the nape of the neck in the posterior compartment and one each in the larynx, vallecula, and the orbit, respectively. The duration of the lesions ranged from one month to seven years. Tumors ranged from 1-15 cm (average 5.9 cm) in size. The need for medical intervention was cosmetic reasons (n=3) and/or obstructive symptoms (n=3).

**Table 1:** Clinical details of the patients M: male; F: female; NED: no evidence of disease; AE: aryepiglottic fold; PFS: pyriform space; MDL: microdirect laryngoscopy; NA: not available

Sr. No	Age/ Sex	Site	Size	Duration	Radiology	Initial Diagnosis	Review Diagnosis	Treatment	Follow-up
1	57/M	Nape of neck	7.5cm	3 years	7.5cm lesion with enhancement and focal necrosis. Adipocytic areas were noted. Favour liposarcoma	FNAC - Inadequate	Spindle cell lipoma	Excision	NED; >6months
2	21/M	Left orbit	-	1 month	32x15x21 mm ill-defined lesion in superior and medial quadrants of left orbit encasing superior and medial rectus muSL/PLe with intraconal extension	Biopsy- Angiomatous lesion	Spindle cell lipoma	Excision	NED; >6 months
3	30/M	Vallecula	1cm	1 month	-	Biopsy- Myxoid liposarcoma	Spindle cell lipoma	Excision	NED; >6 months
4	54/F	Left AE fold	3cm	4 months	2.3 x 1.3 x 1.9 cm enhancing thickening of left supraglottis & PFS	Biopsy- Non -representative	Spindle cell lipoma	Excision	Residual swelling in the left PFS region, residual disease; on observation
5	58/M	Nape of neck	15cm	7 years	An indeterminate grade well-defined mass in the subcutaneous plane of the upper back with macroscopic fat within	None	Spindle cell lipoma	Observation	NA
6	39/M	Nape of neck	3cm	-	-	None	Spindle cell lipoma	Excision	NA

**Figure 1:**
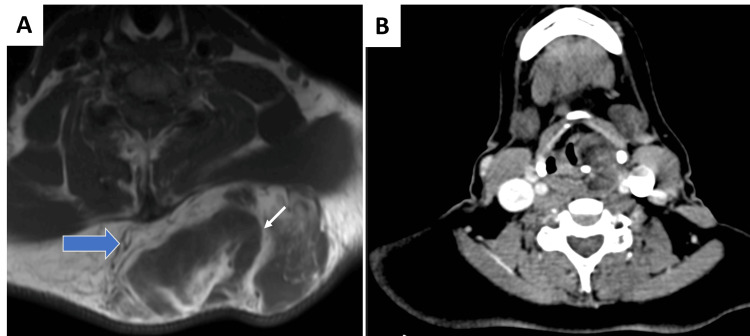
Radiology images A. A well-defined lobulated encapsulated mass (block arrow) is seen in the subcutaneous plane in the upper back. Few hyperintense areas are seen on T1W sequences (white arrow) showing suppression suggestive of fat-containing areas. B. A relatively well-defined hypodense non-enhancing lesion is seen in the left pyriform sinus extending into the para-glottic space at the level of true and false cords without infiltration of the adjacent structures

Radiological information was available in four cases. Case 1 was diagnosed as liposarcoma on radiology due to prominent enhancement and focal necrosis while Case 5 was labeled as an intermediate-grade tumor due to the abundance of non-adipocytic components. Others were diagnosed as mesenchymal neoplasms with the presence of fat within (Figure 1). Biopsy was available in four of the six cases (66.7%) and the diagnoses ranged from non-representative/ inadequate tissue to angiomatous lesion and myxoid liposarcoma. Five out of these six cases (83.4%) were excised with a curative intent while one patient was kept for observation as the patient was unwilling for surgery and the tumor exhibited a stable course. 

**Figure 2:**
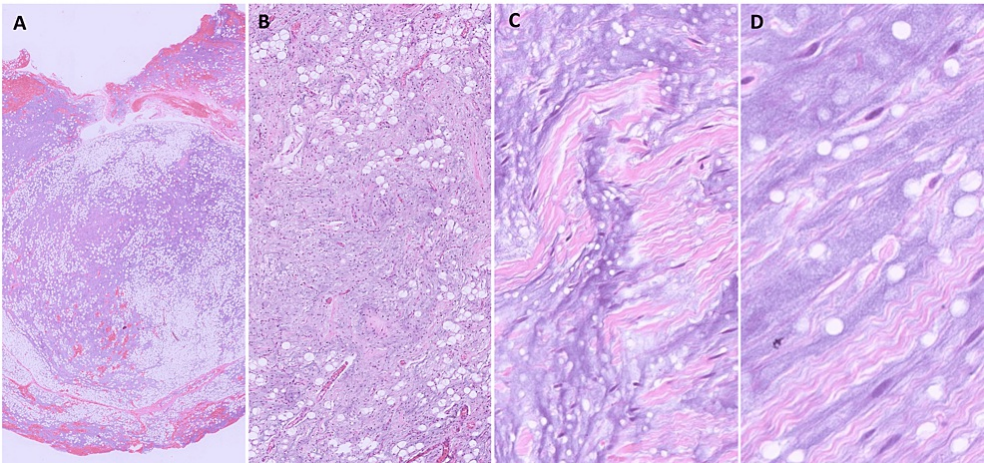
Histomophology of spindle cell/pleomorphic lipoma A. Scanner view showing a well-defined lesion composed of scattered adipocytes in a myxoid stroma. B. (x100 HE) Histology shows a moderately cellular tumor containing plump spindle cells with interspersed fat spaces and delicate capillaries in a prominent myxoid stroma. C & D. (x400 HE) Ropy/ ribbon-like collagen fibrils were seen in the tumor.

**Figure 3:**
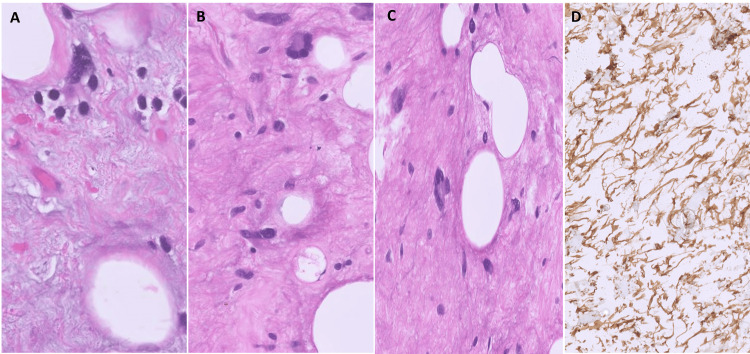
Histomorphology and immunohistochemistry of spindle cell/pleomorphic lipoma A-C. (x400 HE) The tumor showed presence of scattered large, atypical cells with pleomorphic nuclei as well as multinucleated floret-like cells. D. (x400) By immunohistochemistry, the tumor was diffusely and strongly positive for CD34.

Histological examination of all the cases showed a low-grade circumscribed neoplasm composed of a variable amount of bland spindle cells and mature adipocytes; the latter was present in all cases, albeit, to a variable extent. Prominent myxoid stroma was seen in 83.4% (5/6) of the cases. A few of the cases additionally showed variable areas of hyalinization while one case harbored a sclerotic stroma (Figure 2A-2B). Ropy collagen (Figure 2C-2D), prominent vasculature composed of capillary-sized vessels, and the presence of mast cells were noted. Pseudo-angiomatoid spaces were conspicuous in one case (Case 2). Two cases showed the presence of pleomorphic cells, scattered multinucleated stromal giant cells, and floret cells (Figure 3A-3C). None of our cases showed the presence of any metaplastic or heterologous elements. High-grade nuclear features/ significant mitoses including atypical forms or necrosis were not identified in any of these cases. In Case 3 (vallecular), the tumor was completely submucosal with the overlying hyperplastic squamous epithelium of the vallecula. By IHC, all cases showed diffuse positivity for CD34 in the spindle cells (Figure 3D), while S100, SMA, and desmin were negative. Case 4 additionally showed focal weak positivity for MDM2 and p16. 

Three out of these six cases had alternate diagnoses on the initial histopathologic reporting of the biopsies that were subsequently revised to SL/PL after review. Due to the presence of prominent vasculature and diffuse myxoid stroma, Case 2 was initially diagnosed as an angiomatous lesion elsewhere. Similarly, a laryngeal biopsy (Case 4) showing fat and myxoid collagenous stroma was interpreted as non-representative and a repeat biopsy was advised. However, a multidisciplinary joint clinic discussion indicated that the lesion was a submucosal lesion and the radiologist was confident that the biopsy was representative following which CD34 IHC was added and the diagnosis revised to SL/PL. Following this, the patient underwent debulking surgery and was confirmed to be SL/PL on histopathology. Due to the difficult anatomical location, complete resection with a clear margin was not possible with residual disease in the right pyriform sinus region. The patient is now on close follow-up to monitor the increase in the size of the lesion.

## Discussion

SL/PL is a distinct type of lipoma characterized by mature fat, collagen, and spindle cells. They have a marked male predilection (>90%) with an age range of 45-65 years [4]. Our cohort also showed a male-to-female ratio of 5:1 with a mean age of 34.8 years. Male predilection is explained by the frequent detection of androgen receptors in SL/PL [9]. Clinically, SL/PL is slow-growing solitary painless masses with a size range of 2-29 cm, the majority being 3-5 cm [3,4]. The clinical presentation largely depends upon the location of the tumor. Intra-oral or laryngeal tumors often present with dyspnea or dysphagia of short duration whereas those involving the soft tissues of the neck or other sites remain indolent for years despite their large sizes [5]. While the neck, upper back, and shoulder are the characteristic sites [2], presentation at uncommon sites can lead to diagnostic difficulties. Occurrence in the oropharynx and larynx is extremely rare [6-8]. Laryngeal lipomas are extremely rare (less than 150 cases reported in the literature so far) accounting for 0.6% of all benign laryngeal tumors [7,8]. To date, only six cases of SL/PL of the larynx have been reported in the literature (Table 2) [6,7,10-13]. Two cases in our cohort arose in the vallecula and larynx, respectively, and caused diagnostic dilemmas during reporting. 

**Table 2:** Summary of case reports of laryngeal spindle cell lipoma/pleomorphic lipoma M: male; F: female; MDL: microdirect laryngoscopy; AE: aryepiglottic; CO: carbon dioxide

Sr. No	Author	Age/ Sex	Duration	Symptoms	Size	Location	Treatment
1	Nonaka et al., 1993 [10]	68/M	6 months	Dysphonia	2.6cm	Vestibular fold and ventricle	Laryngofissure
2	Nader et al., 2012 [11]	63/M	Several months	Snoring and dyspnea	2.7cm	Left aryepiglottic and glottis	Endoscopic
3	D’Antonio et al., 2013 [12]	65/M	Two weeks	Dysphonia, dyspnea and stridor	-	Large polyp of the left true vocal fold	MDL
4	Kodiyan et al., 2015 [6]	79/F	One year	Dysphonia	3.5cm	Right supraglottis, the arytenoid and aryepiglottic fold	MDL and laser
5	Wolf-Magele et al., 2016 [13]	52/M	-	Pain, stridor, and dyspnea	8cm	Larynx	CO2 laser
6	Azar et al., 2020 [7]	35/M	-	Progressive dyspnea	4cm	Left lateral pharyngeal wall, pyriform sinus and supraglottis	Tracheostomy, MDL and laser
7	Current study, Case 3	30/M	1 month	Dyspnea	1cm	Vallecula	MDL
8	Current study, Case 4	54/F	4 months	Dyspnea	3cm	Left AE fold	Excision

In radiology, the diagnosis of SL/PL is often challenging due to the presence of variable amounts of spindle cells and adipocytic components; and the often present myxoid stroma. At times, it may be difficult to distinguish from liposarcoma or other adipocytic neoplasms or even from other low-grade sarcomas. The presence of intramural fat in radiology is often seen in SL/PL; however, its absence does not rule out the same. The adipose tissue shows low attenuation on CT scans and has a density lower than water (zero or negative Houndsfield units) [14]. SL/PLs are iso-intense to fatty tissue on MRI; however, due to the variable proportion of spindle cells and the myxoid or hyalinised stroma, it may look similar to the muscle [15] Also, the non-adipocytic component of the SL/PL often enhances following the administration of intravenous contrast material [4]. Due to the presence of a variable amount of mesenchymal components in the SL/PL, they can be misdiagnosed as liposarcoma in radiology, and hence a meticulous histopathological examination is required for confirmation [4].

Grossly, these are usually well-circumscribed nodular/globular lesions with sharp margins from the surrounding. The cut surface is often variable depending upon the proportion of adipose tissue and spindle cells. At times, it may be glistening or gelatinous owing to a prominent myxoid stroma [3,4]. Microscopically, SL/PL are well-circumscribed, non-capsulated lesions composed of a triad of mature adipose cells, bland spindle cells, and hyalinized ropy collagen [4,12,16]. The spindle cells are short and elongated with indistinct cell borders and eosinophilic cytoplasm with minimal or no cellular atypia. Multinucleate stromal giant cells and floret cells are seen in the PL cases. Myxoid stroma with conspicuous mast cells is frequent. Hyalinized to sclerotic stroma may be seen. At times, fatty components may be markedly reduced which renders the diagnosis quite challenging [17,18]. Peudoangiomatous pattern with spaces simulating a vascular tumor may be seen. Infrequently, chondroid or osseous metaplasia [19] or rarely, extramedullary hematopoiesis may be seen [20]. Features of malignancy in the form of infiltrative growth, necrosis, pleomorphic lipoblasts, and significant mitotic activity are absent.

PL is closely related to SL in all aspects with both showing CD34 positivity and loss of RB1 expression except for the presence of pleomorphic and multinucleated floret-like giant cells. Therefore, PL and SL are considered two ends of the spectrum of the same entity [8,21].

Other lipomatous tumors frequently need distinction from SL/PL. Fibrolipomas are lipomas with focally increased fibrous tissue. These may show positivity for CD34; however, the presence of spindle cells and often myxoid stroma may help in diagnosing SL/PL [22]. Atypical spindle cell/pleomorphic lipomatous tumors contain spindle cells and adipocytes akin to SL/PL, however, they additionally have lipoblasts, hyperchromatic pleomorphic spindle cells, and an infiltrative tumor front. These are often deep-seated, arise in the extremities and limb-girdle, and display a male-to-female ratio of 3:2. In contrast, SL/PL are superficial, well-circumscribed, and contain ropy collagen along with spindle cells and adipose tissue. On IHC, atypical spindle cell/ pleomorphic lipomatous tumor shows variable positivity for CD34, S100 protein, and desmin, while MDM2 and CDK4 are often negative. On the other hand, SL/PL is consistently positive for CD34 and negative for desmin and S100p [16,23]. Loss of retinoblastoma 1 (*Rb1*) gene expression is seen in 50-70% of cases of atypical spindle cell/pleomorphic lipoma which is also seen in SL/PL and correlates with the deletion of 13q14 harboring the RB1 locus [23,24]. Well-differentiated liposarcoma (WDLS) is another close differential diagnosis. However, deeper location, cytological atypia, retained *Rb1* gene expression, and the presence of *MDM2 *gene amplification in WDLS can distinguish SL/PL from WDLS [3,16]. Pleomorphic liposarcoma is an uncommon subtype of liposarcoma with marked cytological atypia and infiltrative edges with atypical mitosis and necrosis. Although they can show loss of RB1, they usually possess additional complex chromosomal gains and losses unlike SL/PL [25].

Many non-lipogenic tumors also show overlapping features and need to be distinguished from SL/PL. These include nodular fasciitis, peripheral nerve sheath tumors, myofibroblastomas, solitary fibrous tumors, and dermatofibrosarcoma. Immunohistochemical positivity for CD34 and negativity for S100P, SOX10, desmin, smooth muscle actin, and STAT6 favors SL/PL [22,25,26]. Nodular fasciitis (NF) shows zonal maturation with a loose feathery center containing tissue-culture myofibroblasts admixed with lymphocytes and extravasated erythrocytes and peripheral more mature fibroblasts. While histologic features are characteristic, myxoid nodular fasciitis may mimic SL/PL in a biopsy. CD34 is negative in NF while *USP6* gene rearrangements are characteristic of NF [27]. Benign peripheral nerve sheath tumors show bland spindle cells with wavy nuclei and may show prominent myxoid stroma; however, they are diffusely positive for S100P or SOX10. A solitary fibrous tumor (SFT) is a variably cellular tumor composed of spindle cells and thick bands of collagen, and hemangiopericytomatous vascularity. The spindle cells often form whorls around the small capillaries. SFT shows prominent branching and hyalinised blood vessels, unlike SL/PL. A small proportion of SFTs show the presence of adipose tissue and are labeled as lipomatous SFTs. SFT can also show the presence of entrapped adipose tissue. Immunoreactivity for STAT6 excludes the diagnosis of SL/PL [22,25,26]. Dermatofibrosarcoma protuberans (DFSP) is also included in the differentials of SL/PL due to the presence of fascicular spindle cells and myxoid stroma. However, DFSP shows an infiltrative growth pattern with entrapment of adjacent fat rather than the latter being a part of the tumor. Also, DFSP harbors t(17;22) (q22;q13) resulting in COL1A1-PDGFB fusion, which is not seen in SL/PL [22,25,26].

The choice of treatment for SL/PL is excision with a clear margin; irrespective of the site [5]. For laryngeal SL/PL, radical excision of the tumor with an endoscopic approach is the preferred mode of treatment [28,29]. Although recurrences are uncommon, a long-term follow-up is recommended. Recurrences may result secondary to incomplete primary excision of the tumor or it may indicate that the tumor was a WDLS; thus requiring aggressive management of the tumor [11].

Limitations

This is a comprehensive study on SL/PL of the head and neck region. However, there were certain limitations in our study. The smaller sample size is one of the major limitations of our study; however, these tumors are rare and uncommon. Adequate sampling of the tumor is necessary to rule out close differential diagnosis of SL/PL such as the WDLS and atypical spindle cell/pleomorphic lipomatous tumor. Although it is not needed for diagnosis, the lack of IHC staining showing loss of *the RB* gene would have benefited the ruling out of other differential diagnoses of SL/PL. The cases are from a single center that introduces a potential bias related to patient demographics, clinical practices, and diagnostic approaches specific to that institution. Extrapolating findings to broader populations or different healthcare settings should be done cautiously considering this limitation. Prospective studies with larger sample sizes and standardized protocols could provide more robust insights into SL/PL characteristics and management.

## Conclusions

The current series describes cases of SL/PL occurring in the head-neck region including rare locations such as the larynx. SL/PL are otherwise benign tumors with minimal risk of recurrence following resection. Surgeons play a pivotal role in ensuring optimal patient outcomes through meticulous excision, guided by precise pathological assessment. A combination of histopathology along with immunoreactivity for CD34 and loss of the *Rb1* gene supports the diagnosis. Long-term follow-up remains imperative, underscoring the interdisciplinary collaboration essential for navigating the complexities of this intriguing entity in surgical oncology.
